# Enhanced predictive saccade strategies and spatial prediction accuracy in first-person shooter-specialized players

**DOI:** 10.3389/fnsys.2026.1775973

**Published:** 2026-03-09

**Authors:** Ryo Koshizawa, Zdeněk Ledvina, Jakub Pospíšil, Ondřej Peleška

**Affiliations:** 1 College of Economics, Nihon University, Tokyo, Japan; 2 Faculty of Physical Education and Sport, Charles University, Prague, Czechia

**Keywords:** esports, FPS-specialized players, low-beta oscillations, predictive saccades, spatial prediction, supramarginal gyrus (BA40), visuomotorprediction

## Abstract

**Introduction:**

Predictive gaze behavior is essential in fast-paced esport environments; however, the visuomotor and neural mechanisms supporting predictive saccades in competitive first-person shooter (FPS) players remain insufficiently understood. This study investigated whether FPS-specialized players exhibit enhanced predictive saccade strategies compared to individuals without competitive FPS experience.

**Methods:**

Seventeen active gamers were assigned to either an FPS-specialized group (*n* = 6) or a non-FPS group (*n* = 11). Participants performed a target-arrival prediction task in which a parabolically moving target was occluded midway through its trajectory. They were instructed to fixate on the starting point, execute a predictive saccade toward the internally estimated arrival position, maintain fixation, and press a button at their judged arrival time. Position Error (PE) was derived from gaze and button-press data. Low-beta (12–16 Hz) electroencephalography (EEG) activity was extracted using the Hilbert transform and group differences were assessed using time-series statistics and cluster-based permutation testing.

**Results:**

The FPS-specialized group exhibited earlier emergence of predictive gaze shifts toward the anticipated arrival position and demonstrated substantially smaller spatial prediction errors, including reduced PE values. These behavioral advantages were accompanied by increased low-beta activity in right Brodmann area (BA) 7, left BA40, and left BA6—regions, associated with spatial prediction, visuomotor integration, and predictive motor planning.

**Conclusion:**

These findings suggest that competitive FPS experience cultivates a coordinated visuomotor prediction system that supports earlier initiation and improved accuracy of predictive saccade behavior.

## 1 Introduction

In many traditional sports, athletes are considered to be approaching the limits of physical performance. As a result, recent scientific and technological advances have shifted attention toward the potential for further improvement through cognitive enhancement (Campbell et al., 2018). Esports have rapidly emerged as a competitive domain requiring high-level cognitive abilities and have attracted growing interest from neuroscience and cognitive science researchers (Campbell et al., 2018). Although esports differ from traditional sports in that they involve seated interaction with visual displays rather than whole-body movements, they impose similarly demanding perceptual, cognitive, and motor requirements. High-level performance requires rapid processing of complex visual stimuli, precise bimanual motor actions, and decision-making under strict temporal constraints. Players must flexibly allocate attention across multiple onscreen elements, track peripheral cues such as mini-maps and status indicators, and simultaneously execute finely controlled actions to avoid opponents and hazards (Campbell et al., 2018).

From a neurocognitive standpoint, esports share key mechanisms with traditional sport expertise. Neuroimaging studies have shown that neural activity in regions such as the posterior parietal cortex (PPC) decreases as skill level increases, reflecting more efficient allocation of cognitive resources among highly trained players (Bavelier et al., 2012a, 2012b; Campbell et al., 2018). Similarly, action video game players (VGPs) exhibit reduced cortical activation in motion-sensitive regions (middle temporal; MT) during the processing of moving distractors, suggesting enhanced filtering of irrelevant information before it reaches higher-order areas (Bavelier et al., 2012a, 2012b). Crucially, the core demands of esports—selective attention, heightened sensitivity to dynamic stimuli, and predictive processing under severe time pressure—are functionally comparable to those observed in traditional ball sports. For example, in baseball or cricket, batters must anticipate the trajectory and timing of a pitch within a few 100 milliseconds between release and its arrival at the plate. This requirement is closely paralleled in esports where players must predict opponents’ actions and rapidly changing environments with millisecond-level precision (Campbell et al., 2018; Green and Bavelier, 2012).

These parallels suggest that insights from traditional sports neuroscience—particularly those addressing predictive visual strategies—may be transferable to esports research and vice versa. Moreover, because esports are performed in relatively stationary and controlled environments, they provide opportunities to collect high-quality neurophysiological data with fewer motion-related confounders than full-body athletic performance. This aligns with prior arguments positioning esports as a valuable domain for studying high-performance cognition (Campbell et al., 2018). Collectively, these perspectives support the view that esports represent a cognitively rich environment in which predictive visual strategies are central to performance optimization.

Previous research has consistently demonstrated that general gaming experience is associated with enhanced visuospatial and attentional processing. Diverse behavioral paradigms—including flanker tasks, enumeration tasks, useful-field-of-view testing, attentional blink paradigms, and stimulus–response mapping—have consistently shown that VGPs outperform non-players across core perceptual and attentional domains (Castel et al., 2005; Green and Bavelier, 2003). These advantages include enhanced visual sensitivity (West et al., 2008), reduced task-switching costs and greater cognitive flexibility (Colzato et al., 2010), and superior endogenous attentional control (Chisholm et al., 2010). These gains may persist for months following reduced gameplay, indicating stable neurocognitive adaptation (Feng et al., 2007). Action VGPs also demonstrate finer spatial resolution, higher contrast sensitivity, and expanded visual fields relative to non-players (Buckley et al., 2010; Green and Bavelier, 2007; Li et al., 2009).

Neurophysiological findings, however, are more heterogenous. Expert VGPs exhibit faster behavioral responses, reduced hemispheric asymmetries in motor responses, and shorter visual N1 latencies, an electrophysiological marker of early perceptual processing (Latham et al., 2013). N1 latency correlates with reaction time, suggesting that accelerated neural processing contributes to superior visuospatial performance. Similarly, Mishra et al. (2011) reported that VGPs suppress irrelevant information streams more effectively, reflected in reduced steady-state visual evoked potentials (SSVEPs) to distractors, and larger P300 amplitudes to targets under high perceptual load. Complementary functional Magnetic Resonance Imaging (fMRI) studies have shown that action VGPs rely less on frontoparietal attentional resources under high perceptual load, consistent with more efficient and automated allocation of attention (Bavelier et al., 2012a, 2012b).

Comprehensive reviews have synthesized these findings, concluding that action video games induce enhancements in perception and attentional control across multiple temporal scales—from milliseconds to minutes (Green and Bavelier, 2012). More recent neuroimaging studies suggest that these behavioral advantages reflect network-level neuroplastic refinement rather than isolated regional changes, indicating reorganization of the visuomotor and attentional systems in action VGPs (e.g., Cahill and Dhamala, 2025). Taken together, this evidence positions esports as a cognitively demanding domain in which expertise is strongly associated with attentional and executive control.

However, despite the expanding literature, much of this work has emphasized reactive performance measures and stimulus-driven attentional paradigms, leaving open the question of how such network-level adaptations support predictive visuomotor strategies during continuous dynamic tasks that closely resemble real-world performance. Esport performance extends beyond basic attentional allocation; it requires sophisticated strategic reasoning, dynamic gaze control, and predictive cognitive processing. In first-person shooter (FPS) games, highly trained players typically avoid prolonged fixation on a single opponent. Instead, they employ brief fixation durations and wider gaze distributions to scan the environment, maintain threat monitoring, and anticipate unfolding events (Jeong et al., 2025). These dynamic gaze behaviors reflect predictive processing and are tightly coupled to competitive performance.

To address the limited focus on predictive gaze strategies in esports, our prior investigations established foundational evidence for predictive visuomotor mechanisms in target-arrival prediction tasks. Koshizawa et al. (2024) demonstrated that predictive saccades were associated with increased low-beta activity in visual and motor regions and with improved prediction accuracy. However, that study did not examine the temporal precision of saccade execution. Since predictive saccades are advantageous only when executed early enough to influence subsequent visuomotor processing, characterizing their temporal features is critical. Extending this work, Koshizawa et al. (2025) compared early versus late predictive saccades and showed that earlier execution was associated with earlier accurate gaze positioning and stronger low-beta activity in regions corresponding to the frontal eye field and MT area. Taken together, these findings suggest that accurate estimation of motion parameters may facilitate earlier initiation and improved accuracy of predictive saccades—mechanisms likely relevant to high-level esport performance.

Building on this framework, the present study investigates how predictive saccade strategies are employed by an FPS-specialized group compared with participants without competitive/tournament-level FPS involvement, irrespective of other gaming genres. Specifically, we focused on two dimensions: (1) the timing of predictive saccade execution and (2) the spatial accuracy of arrival position prediction. We hypothesized that participants with competitive FPS experience would initiate predictive saccades earlier and with greater endpoint accuracy, and that these behaviors would be associated with increased low-beta (12–16 Hz) activity in visuomotor regions.

## 2 Materials and methods

The experimental design of the present study was adapted from the paradigm introduced by Koshizawa et al. (2025), who examined electroencephalography (EEG) activity associated with predictive saccade strategies. Unlike the previous study, the present work introduced a group-based comparison between FPS-specialized and non-FPS participants. This modification enabled investigation of whether predictive saccade strategies and their associated neural activity differ across varying levels of competitive FPS experience.

### 2.1 Participants

Seventeen healthy adults (mean age: 22.82; 15 men, 2 women) participated in the study. Only participants whose eye movements could be reliably recorded using the Gazefinder® infrared eye-tracking system (JVC Kenwood, Kanagawa, Japan) were included in the analysis. All participants had normal or corrected-to-normal vision and reported no history of psychological, psychiatric, or neurological disorders.

Participants were active VGPs recruited from local universities and gaming communities. Those who identified FPS titles (e.g., VALORANT, Counter-Strike, Call of Duty) as their primary gaming domain and reported participation in online or offline tournaments were classified as the FPS-specialized group (*n* = 6). The remaining participants, who did not report competitive or tournament-level FPS experience, were assigned to the non-FPS group (*n* = 11). Within the FPS-specialized group, all participants reported FPS titles (e.g., *VALORANT*, *Counter-Strike*, and *Call of Duty*) as their primary gaming domain and had experience in organized competitive play, including participation in online or offline tournaments, local or school-level competitions, or higher-level national tournaments; some participants had experience at semi-professional or professional levels. In contrast, the non-FPS group was heterogeneous in composition. This group primarily consisted of individuals who either competed at higher levels in non-FPS genres (e.g., national-level tournaments in non-FPS titles) or engaged in FPS games casually (e.g., ranked matches for recreation without tournament participation). Importantly, participants in the non-FPS group who reported tournament level competition did not compete in FPS titles, whereas those who played FPS games did so exclusively at the casual, noncompetitive level. All participants were right-handed.

Written informed consent was obtained prior to participation. The study protocol was reviewed and approved by the Ethics Committee of the Faculty of Physical Education and Sport, Charles University (Approval ID: 96/2025), and adhered to the principles of the Declaration of Helsinki. The project, titled “Visual Processing and Saccadic Strategies in eSports Players: An EEG and Eye-Tracking Study”, was conducted between August and October 2025. Participants were instructed to abstain from alcohol and drug use on the day prior to testing to minimize potential confounding factors.

### 2.2 Apparatus

#### 2.2.1 Eye position

Eye position was recorded using the Gazefinder® infrared eye-tracking system (JVC Kenwood, Kanagawa, Japan). Stimuli were presented on a 19-inch monitor (1280 × 1024 pixels) equipped with integrated infrared cameras. The monitor was positioned approximately 60 cm from the participant’s eyes, corresponding to a visual angle of 35.66° horizontally and 28.86° vertically. Gaze coordinates (X, Y) were sampled at 50 Hz. A standard 5-point calibration procedure was completed prior to each experimental session. Only participants whose eye positions could be reliably captured during calibration and task execution were retained for analysis.

#### 2.2.2 Electroencephalography

EEG signals were recorded using a PolymatePro MP6100 system (Miyuki Giken, Tokyo, Japan) with Ag/AgCl electrodes placed according to the international 10–20 system (10–10 extensions included). Recordings were obtained from the following electrode sites: FC1, FC2, FC5, FC6, CP1, CP2, CP5, CP6, P3, P4, P7, P8, O1, O2, FCz, Cz, Pz, POz, and Oz. The reference electrode was positioned at CPz, with AFz serving as ground. Data were sampled at 1000 Hz, and electrode impedances were maintained below 20 kΩ. To synchronize EEG recordings with stimulus presentation, a photosensor (Miyuki Giken, Tokyo, Japan) was attached to the stimulus monitor. While the apparatus configuration was broadly consistent with previous predictive saccade studies (e.g., Koshizawa et al., 2025), the present experiment incorporated one key modification: the use of a PolymatePro system with a customized electrode montage optimized for visuomotor processing.

### 2.3 Task

The experimental task was adapted from Koshizawa et al. (2025), who examined EEG activity associated with saccade strategies during target occlusion. In contrast to the earlier work, the present study compared neural activity between the FPS-specialized and non-FPS groups, enabling a direct investigation of domain-related visuomotor expertise.

**Figure 1:**
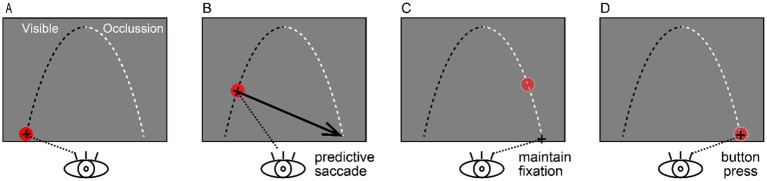
Experimental Paradigm. A red target traveled along a parabolic trajectory from the lower left to the lower right of the display. The first half of the trajectory (0–2.3 s) was visible, and the second half (2.3–4.6 s) was occluded. Participants were instructed to **(A)** fixate the starting position, **(B)** execute a predictive saccade toward the internally predicted arrival position once they computed their prediction, **(C)** maintain fixation at that predicted location, and **(D)** press a response button at their judged arrival time.

As illustrated in Figure 1, participants were instructed to predict both the arrival position and timing of a target moving along a parabolic trajectory from the lower-left to the lower-right area of the display. The trajectory simulated gravitational motion, with the landing point positioned at the same vertical level as the launch position. To prevent habituation, three trajectory conditions—CLOSE, MID, and DISTANT—were randomized across trials. These trajectories differed in horizontal distance, initial velocity, and launch angle and were computed assuming constant gravitational acceleration (9.81 cm/s^2^) with no air resistance.

#### 2.3.1 Target characteristics

The moving target was a red circle (RGB: 255, 0, 0; radius: 0.38°) presented against a gray background (RGB: 128, 128, 128). Each trajectory lasted 4.6 s. During the first 2.3 s, the target remained visible; during the remaining 2.3 s, its trajectory was occluded using a gray overlay of the same chromatic value. Across the CLOSE, MID, and DISTANT conditions, trajectory parameters were systematically adjusted to vary the final landing positions (see Table 1).

**Table 1:** The launch angles and velocities for each target type.

Target type	Initial velocity (°/s)	Launch angle (°)	Highest point (x, y) (cm)	Final position (x, y) (cm)
Close	23.28	75.96	(12.98, 26.00)	(25.97, 0.12)
Mid	23.33	75.45	(13.48, 26.00)	(26.97, 0.12)
Distant	23.39	74.93	(13.98, 26.00)	(27.97, 0.12)

#### 2.3.2 Procedure

Each trial consisted of three sequential phases: (1) Fixation phase: A red circle appeared at the launch position for 2.4–3.6 s (randomized). (2) Motion phase: The target moved along a parabolic trajectory for 4.6 s, with the first half visible and the second half occluded. (3) Response phase: Participants were instructed to: a. shift their gaze to the predicted arrival position as soon as they formed their prediction; b. maintain fixation at that predicted location until they estimated that the target had arrived; and c. press a response button with their right thumb at their judged arrival time.

Before the main experiment, participants completed a short practice block using a mirrored trajectory (right-to-left motion). The main task consisted of six blocks of 30 randomized trials, with approximately 2-min rest intervals between blocks.

### 2.4 Data analysis

Trials were excluded from analysis if they met any of the following criteria: (1) loss of eye-position data caused by blinks occurring between target-motion onset and the participant’s button-press response; (2) signal dropouts or tracking errors in the eye-tracking system; or (3) EEG artifacts exceeding ±100 μV in any channel within the analysis window (−200 to +2,300 ms relative to target onset), following standard artifact rejection procedures (Park and Donaldson, 2019). Trial exclusion was performed in a stepwise manner. First, trials containing data loss due to blinks or signal dropout occurring between target motion onset and the participant’s button-press response were excluded from all analyses. EEG artifact rejection was subsequently applied only to the remaining trials. In the non-FPS group, an average of 76.64 trials per participant (SD = 37.14) were excluded due to blink-related data loss or signal dropout, and an additional 12.36 trials (SD = 21.75) were excluded due to EEG artifacts exceeding ±100 μV, resulting in a total of 89.00 excluded trials per participant on average (SD = 35.17). In the FPS-specialized group, an average of 49.00 trials per participant (SD = 44.46) were excluded due to blink-related data loss or signal dropout, and an additional 8.00 trials (SD = 9.06) were excluded due to EEG artifacts, resulting in a total of 57.00 excluded trials per participant on average (SD = 39.52). EEG analyses focused on the visually guided phase of the task (0–2,300 ms), prior to occlusion onset. Data recorded during the occluded portion of the trajectory (2,301–4,600 ms) were excluded because this period frequently contained button-press responses and associated motor preparation activity that was not directly related to visuospatial prediction. In contrast, eye-position data were analyzed until the participant’s individual button-press timing to capture gaze dynamics associated with predictive fixation behavior.

Although three trajectory conditions (CLOSE, MID, DISTANT) were included to increase trial variability and minimize habituation, they were not analyzed separately. Instead, trials across all trajectory conditions were pooled to maximize the number of artifact-free trials and enable a unified analysis of visuomotor and neural dynamics. The primary aim of the present study was to compare the temporal characteristics of predictive saccade strategies between the FPS-specialized and non-FPS groups.

#### 2.4.1 Eye position

Behavioral performance was quantified using Position Error (PE), defined as the Euclidean distance between the participant’s predicted endpoint (gaze position at the button press) and the actual final target position. This measure reflects the spatial accuracy of endpoint prediction.

A secondary temporal measure, Response Error (RE), was calculated as the unsigned difference between the participant’s response latency and the true target landing time (4.6 s). Smaller RE values indicate more accurate temporal prediction.

To characterize the continuous evolution of prediction, time-series PE was calculated at each 20-ms sampling interval (50 Hz), producing a temporal trajectory of PE values from 0 to 4.60 s. Raw gaze coordinates (X, Y) were retained for supplementary analyses related to gaze strategies. For clarity, raw eye position data (X, Y) were analyzed and visualized in screen-centered pixel coordinates to preserve the original spatial resolution of gaze trajectories over time. In contrast, PE was converted from pixels to degrees of visual angle based on the viewing distance and screen geometry, allowing quantitative comparison of spatial prediction accuracy across participants. This distinction reflects the different analytical purposes of gaze visualization and error quantification. Together, these behavioral metrics enabled a detailed examination of how predictive accuracy unfolded over time and differed between the two competitive groups.

#### 2.4.2 Electroencephalography

EEG data were processed using the EMSE Suite v5.6 (Cortech Solutions Inc.). The EEG signals were first band-pass filtered between 1 and 40 Hz to remove low-frequency drift and high-frequency noise. This broadband filtering step facilitates stable preprocessing and reliable identification of channels or trials containing large-amplitude artifacts.

The filtered signals were subsequently referenced to a common average reference (CAR). Although CAR is often considered optimal for high-density montages, several studies have demonstrated that it is an appropriate reference scheme for 20–32 channel systems with broadly distributed scalp coverage (Hu et al., 2018; Lei et al., 2014). Given that the present 20-channel montage was positioned according to the international 10–20 system with 10–10 extensions, the use of CAR was considered methodologically justified.

EEG epochs were segmented relative to stimulus onset to characterize anticipatory and predictive neural processes during visually guided motion tracking rather than neural activity time-locked to individual eye movements.

Artifact rejection was performed using an amplitude-based criterion. EEG epochs exceeding ±100 μV (peak-to-peak) were identified as containing large-amplitude artifacts such as blinks or muscle activity and were excluded from further analysis. Independent component analysis (ICA) and electrooculography (EOG)-based regression analyses were not performed. Given the relatively limited number of trials per participant and the presence of task-related systematic eye movements, ICA-based correction carries the risk of component misclassification and overcorrection, potentially distorting low-frequency and beta-band neural signals.

For the oscillatory analysis, emphasis was placed on the low-beta frequency range. Although definitions of the low beta band varies across studies (e.g., 8–15, 12–24, or 13–30 Hz), prior research has shown that neural activity within the 13–15 Hz range is particularly sensitive to attentional modulation and visuomotor prediction (Donner et al., 2007; Spironelli et al., 2008; Spitzer and Blankenburg, 2011). This frequency range is also consistent with the band identified in our previous studies examining predictive visuomotor strategies (Koshizawa et al., 2024, 2025). To ensure that the target oscillatory activity was not attenuated near filter boundaries, a slightly expanded bandpass range of 12–16 Hz was applied. This adjustment accounts for the fact that cutoff frequencies fall within the transition band, where signal power is typically reduced by approximately −3 dB (i.e., ~70% amplitude retention), consistent with standard digital filter characteristics (Widmann et al., 2015). Expanding the passband by 1 Hz on either side allowed effective preservation of the target 13–15 Hz signal while minimizing distortion.

Following narrowband filtering, the analytical signal was computed using the Hilbert transform (Freeman, 2007). Instantaneous log-transformed amplitude envelopes within the low-beta band were then derived and used to quantify time-resolved beta-band modulation associated with predictive visuomotor behavior.

### 2.5 Statistical analysis

#### 2.5.1 Eye position

To evaluate group differences in gaze performance between the FPS-specialized and non-FPS groups, statistical analyses were conducted on four parameters: (1) PE at response — the Euclidean distance between the predicted endpoint (gaze position at button press) and the actual target landing point; (2) RE — the absolute deviation between the participant’s response time and the true target landing time (4.6 s); (3) PE over time (0–4.60 s) — a time-series measure of prediction accuracy across the full trajectory period; and (4) Eye position (X–Y coordinates, 0–4.60 s) — the recorded spatial location of gaze throughout the trial.

Participants were categorized into two groups: FPS-specialized (*n* = 6) and non-FPS (*n* = 11). For PE at response and RE, independent-samples *t*-tests were performed to compare mean performance between groups. For the time-series measures (PE over time and eye position), pointwise independent-samples *t*-tests were computed at each 20-ms sampling interval.

Equality of variance was assessed using Levene’s test. When Levene’s test suggested a trend toward heteroscedasticity (0.05 ≤ *p* < 0.10), Welch’s t-test was applied as a conservative alternative.

Given the large number of comparisons in the time-series data, Benjamini–Hochberg false discovery rate (FDR) correction was applied to control Type I error. Uncorrected *p*-values were reported for transparency; however, all effects that were significant before correction also remained significant after FDR adjustment.

All analyses were performed using IBM SPSS Statistics 29, with the significance threshold set to *p* < 0.05. Welch’s correction was used only when Levene’s test indicated potential variance inhomogeneity.

#### 2.5.2 Electroencephalography

Group differences in low-beta EEG power (12–16 Hz) were examined to assess neural activity associated with predictive visuomotor processing. EEG epochs were time-locked to stimulus onset rather than saccade onset, reflecting the primary aim of characterizing anticipatory and predictive neural processes during visually guided motion tracking rather than neural activity associated with the execution of individual eye movements. This stimulus-locked approach emphasizes group-level temporal modulation of low-beta amplitude envelopes and reduces sensitivity to transient, trial-specific oculomotor events. Accordingly, analyses focused on sustained differences in oscillatory amplitude dynamics across conditions, rather than on the precise timing of individual saccades or microsaccadic events.

Because the EEG data were time-continuous and event-related, nonparametric permutation testing was applied at each 1-ms time point across all 20 scalp electrodes, following established recommendations for time-series EEG analysis (Ally and Budson, 2007; Greenblatt and Pflieger, 2004). For each comparison, 1,000 permutations were used to construct a null distribution. Cluster-based permutation correction was applied to control for multiple comparisons and spatial dependencies among neighboring electrodes. Clusters were formed using an initial uncorrected threshold of *p* < 0.05 at the electrode level, with spatial adjacency defined according to the standard electrode neighborhood implemented in EMSE, reflecting physical proximity within the international 10–20/10–10 system. Cluster-level significance was assessed using the maximum cluster-level statistic (i.e., the sum of t-values within each cluster), which is a widely adopted and validated approach in visuomotor EEG research (Koshizawa et al., 2022, 2024, 2025).

Consistent with prior work (Naccache et al., 2014), effects were considered statistically reliable if they met both of the following criteria: (1) *p* < 0.05 at the cluster level, and (2) persistence for more than 10 ms, thereby reducing the likelihood that transient noise-driven fluctuations would be interpreted as meaningful effects. Topographical maps were generated to visualize the spatial distribution of significant group differences, with values averaged across contiguous significant time windows.

## 3 Results

### 3.1 Eye position

**Figure 2:**
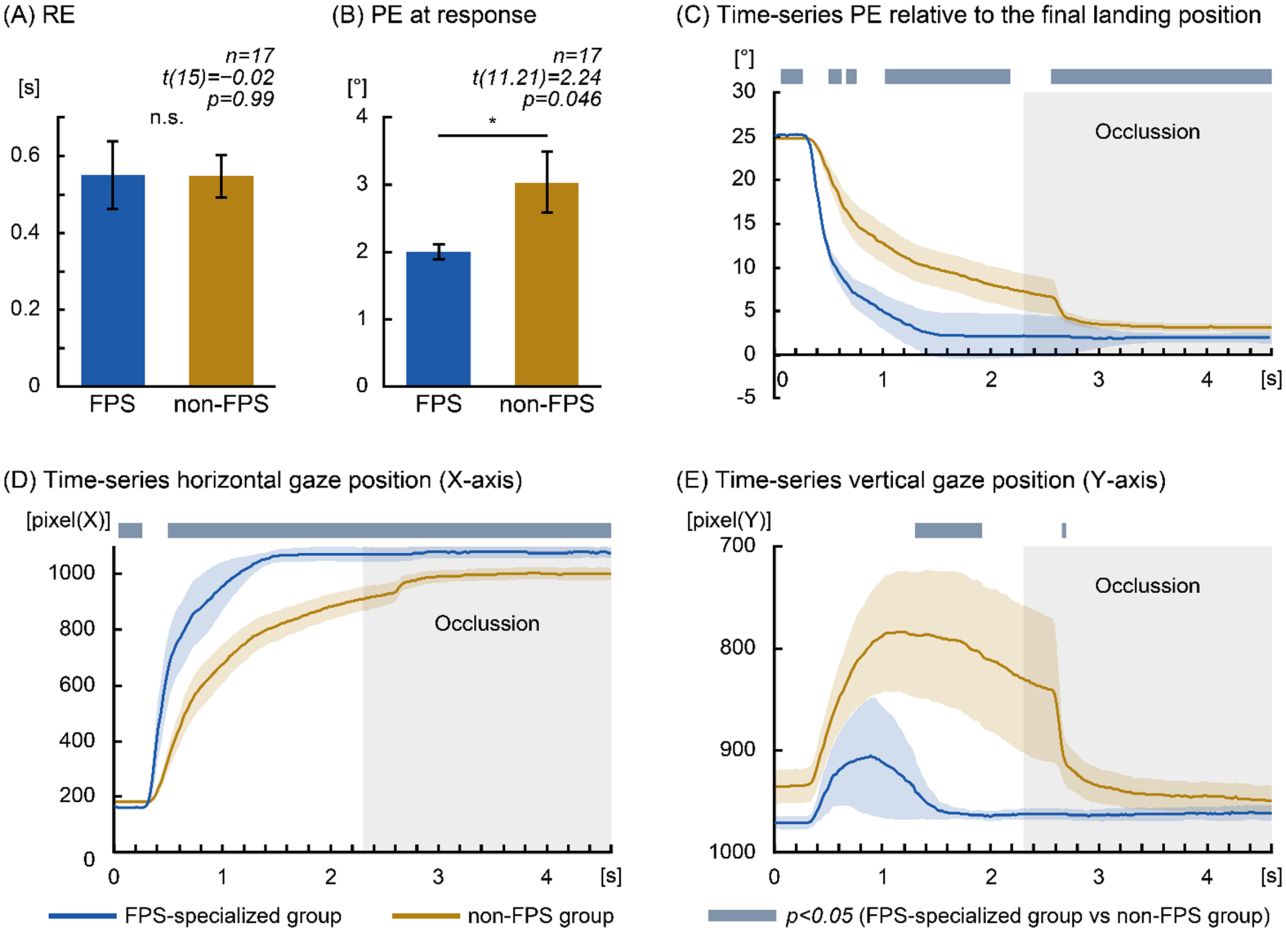
Eye-position–based prediction performance in the FPS-specialized and non-FPS groups. **(A)** Response Error (RE). No significant group difference was observed (“n.s.”). Error bars represent ±1 Standard Error of the Mean (SEM). **(B)** Position Error (PE) at response. The FPS-specialized group demonstrated significantly smaller PE. Error bars represent ±1 SEM. PE is expressed in degrees of visual angle (°). **(C)** Time-series PE relative to the final landing position. PE is expressed in degrees of visual angle (°). **(D)** Time-series horizontal gaze position (*X*-axis). Gaze position is expressed in pixels. **(E)** Time-series vertical gaze position (*Y*-axis). Gaze position is expressed in pixels. Blue traces denote the FPS-specialized group; orange traces denote the non-FPS group. In panels **(C–E)**, the *x*-axis indicates time from target onset (0–4.6 s). The target was visible during the first half of the trajectory (0–2.3 s) and occluded thereafter (2.3–4.6 s). Shaded blue bars denote significant intervals (*p* < 0.05, independent-samples *t*-tests). False Discovery Rate (FDR) correction was applied only to verify cluster stability; unadjusted *p*-values are reported.

The comparison of RE revealed no significant difference between the FPS-specialized group (0.55 ± 0.09 s) and the non-FPS group (0.55 ± 0.06 s) [Levene’s test: *F* (1,15) = 0.29, *p* = 0.60; t (15) = −0.02, *p* = 0.99, Cohen’s d = −0.01]. The mean difference in RE was −0.002 s, with a 95% confidence interval spanning zero (95% confidence intervals of [−0.21 s, 0.21 s]), indicating no reliable group difference (Figure 2A).

In contrast, PE at response differed significantly between the groups. The FPS-specialized group demonstrated smaller PE values (2.00 ± 0.11°) compared with the non-FPS group (3.04 ± 0.45°). As Levene’s test approached significance [F (1,15) = 4.18, *p* = 0.059], Welch’s t-test was applied. The group difference remained significant [t (11.21) = 2.24, *p* = 0.046, Cohen’s d = 0.85]. The mean difference in PE at response was 1.04°, with 95% confidence intervals of [0.02°, 2.06°] (Figure 2B).

Time-series analysis of PE relative to the final landing position is shown in Figure 2C. The FPS-specialized group demonstrated significantly reduced PE compared with the non-FPS group across multiple temporal intervals following target onset: 0.50–0.62 s (*p* < 0.05), 0.66–0.76 s (*p* < 0.05), 1.02–2.18 s (1.02–1.24, 1.82–2.18 s: *p* < 0.05, 1.26–1.80 s: *p* < 0.01), and 2.56–4.60 s (2.56–2.64, 2.84–4.60 s: *p* < 0.05, 2.66–2.82 s: *p* < 0.01). In contrast, the non-FPS group showed significantly reduced PE compared with the FPS-specialized group during 0.06–0.26 s after target onset (*p* < 0.05).

Time-series analysis of horizontal gaze position (Figure 2D) revealed significantly greater rightward gaze displacement in the FPS-specialized group 0.50 target onset and persisting until 4.60 s (0.50–1.24, 1.94–4.60 s: *p* < 0.05, 1.26–1.38 and 1.68–1.92 s: *p* < 0.01; 1.40–1.66 s: *p* < 0.001). The non-FPS group exhibited greater rightward gaze displacement during 0.04–0.26 s (*p* < 0.05).

Similarly, the time-series analysis of vertical gaze position (Figure 2E) demonstrated that the FPS-specialized group maintained significantly lower (more downward) gaze positions relative to the non-FPS group during 1.30–1.92 s and 2.66–2.70 s (*p* < 0.05).

Collectively, these findings indicate that although the two groups showed comparable temporal prediction accuracy (RE), the FPS-specialized group demonstrated notably greater spatial prediction accuracy, reflected by reduced PE and earlier rightward and downward gaze transitions.

### 3.2 Electroencephalography

To evaluate group differences in low-beta EEG activity, non-parametric permutation testing was performed at each electrode during the visible trajectory period (0–2.3 s). The analysis focused on the 12–16 Hz frequency range, with instantaneous log-amplitude values extracted using the Hilbert transform.

**Figure 3:**
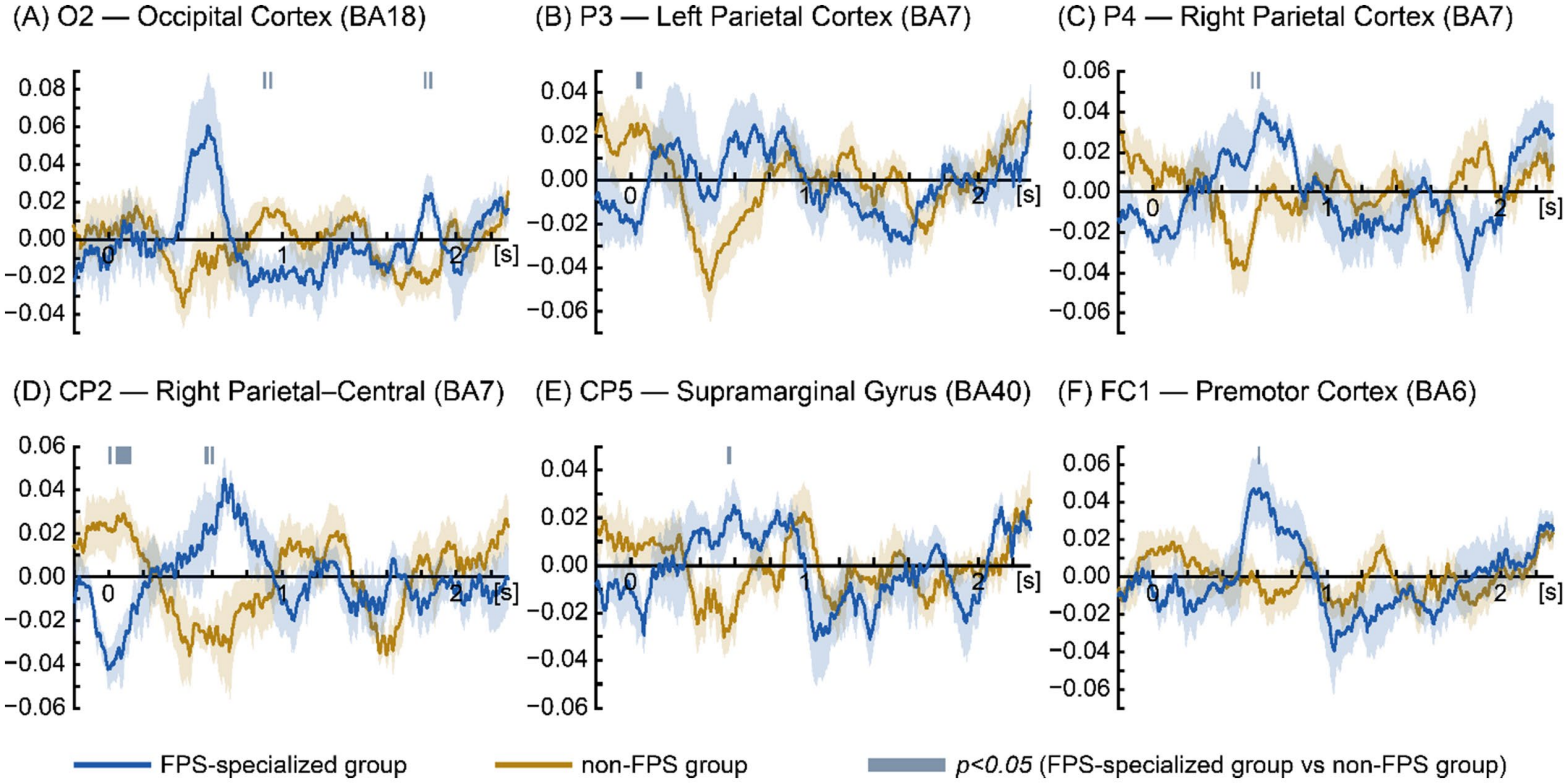
Time-series low-beta (12–16 Hz) EEG activity differences between FPS-specialized and non-FPS groups. Time-series plots display electrodes with significant between-group effects during the visible trajectory period (0–2.3 s). Instantaneous low-beta amplitudes were extracted using the Hilbert transform and represented log-transformed analytic signal envelopes. Panels are ordered from posterior to anterior: **(A)** O2 — Occipital Cortex (BA18). **(B)** P3 — Left Parietal Cortex (BA7). **(C)** P4 — Right Parietal Cortex (BA7). **(D)** CP2 — Right Parietal–Central (BA7). **(E)** CP5 — supramarginal gyrus (BA40). **(F)** FC1 — premotor cortex (BA6). Blue traces indicate FPS-specialized group; orange traces indicate non-FPS group. The *x*-axis represents time from target onset. Shaded blue bars denote significant intervals (*p* < 0.05, non-parametric permutation testing).

Time-series comparisons revealed multiple significant differences between the groups (Figure 3). The FPS-specialized group showed significantly greater low-beta activity at: CP5: 0.554–0.579 s (*p* < 0.05); CP2: 0.554–0.576 s and 0.589–0.607 s (*p* < 0.05); P4: 0.567–0.579 s and 0.602–0.619 s (*p* < 0.05); FC1: 0.603–0.615 s (*p* < 0.05); O2: 1.814–1.830 s and 1.843–1.862 s (*p* < 0.05). Conversely, the non-FPS group exhibited significantly greater activity at: CP2: 0–0.018 s, 0.043–0.090 s (*p* < 0.05), 0.091–0.097 s (*p* < 0.01), and 0.098–0.131 s (*p* < 0.05); P3: 0.034–0.065 s (*p* < 0.05); and O2: 0.890–0.908 s and 0.925–0.941 s (*p* < 0.05).

Figure 3 presents only the electrodes showing significant group differences, arranged from posterior to anterior, in the following order: O2, P3, P4, CP2, CP5, FC1.

**Figure 4:**
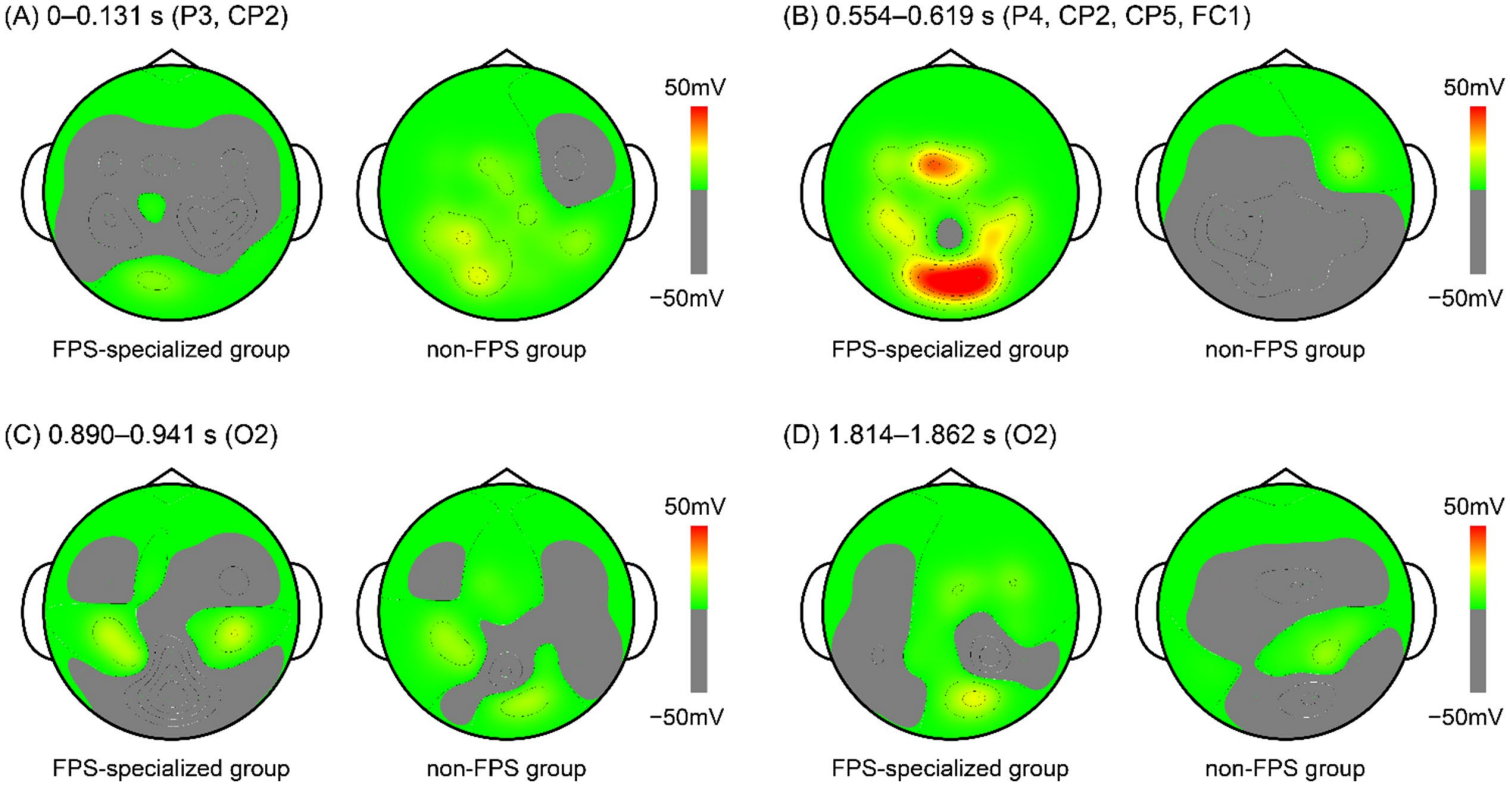
Topographical distribution of group differences in low-beta (12–16 Hz) EEG activity. Scalp topographies display spatial patterns of low-beta activity averaged within the four representative temporal windows identified in the permutation analysis: **(A)** 0–0.131 s (P3, CP2): Early posterior–parietal window showing greater activity in the non-FPS group. **(B)** 0.554–0.619 s (P4, CP2, CP5, FC1): Mid-latency parietal–premotor window showing greater activity in the FPS-specialized group. **(C)** 0.890–0.941 s (O2): Early occipital window showing greater activity in the non-FPS group. **(D)** 1.814–1.862 s (O2): Late occipital window showing greater activity in the FPS-specialized group.

The spatial distribution of significant group differences is shown in the topographical maps (Figure 4). Because several significant time intervals overlapped across electrodes within each functional stage, representative time windows were defined by integrating consecutive or spatially adjacent clusters of significant low-beta differences This approach allowed visualization of stable spatial patterns rather than isolated brief intervals.

Four functional windows were identified. The early window (0–131 ms) reflected significant activity in the non-FPS group at P3 and CP2 (Figure 4A). The mid-latency window (0.554–0.619 s) captured significant intervals in the FPS-specialized group across P4, CP2, CP5, and FC1, consolidating consecutive clusters across these electrodes (Figure 4B). The early occipital window (0.890–0.941 s) showed greater activity at O2 in the non-FPS group (Figure 4C). Finally, the late occipital window (1.814–1.862 s) reflected corresponding significant activity at O2 in the FPS-specialized group (Figure 4D).

These four representative patterns summarize major temporal stages at which group differences emerged and highlight distinct neural profiles associated with visuomotor prediction.

## 4 Discussion

### 4.1 Eye position

Before interpreting gaze dynamics, it should be noted that the eye-tracking system operated at a sampling rate of 50 Hz, which does not allow direct estimation of precise saccade onset latency or fine-grained oculomotor timing. Accordingly, the present analyses focused on group-level predictive gaze dynamics and saccade-related strategies inferred from gaze position and PE time courses, rather than on precise saccade onset metrics.

#### 4.1.1 Spatial and temporal prediction metrics

In the present study, no significant group differences were observed in RE, whereas a clear group difference emerged in PE at response, with the FPS-specialized group exhibiting markedly smaller spatial deviations. This pattern aligns with our previous findings (Koshizawa et al., 2024), in which participants were grouped based on spatial prediction performance. In that study, PE at response reliably distinguished groups, whereas RE did not differ. Thus, the current findings replicate a consistent dissociation previously observed: no difference in temporal accuracy, but a selective advantage in spatial prediction.

Fast response execution has been identified as an important contributor to FPS performance (e.g., Jeong et al., 2025). Supporting this, a systematic review by Toth et al. (2020) reported that action VGPs outperformed non-players in approximately 89% (25/28) of attention-related measures, including reaction time. These findings suggest that sustained engagement in VGPs, including FPS titles, can enhance perceptual–motor responsiveness.

However, the task in the present study did not emphasize reaction speed. Instead, participants were required to produce a motor response at an appropriately estimated moment, making the task a temporal coordination task rather than a simple reaction-time task. The majority of prior FPS research has examined reactive performance (e.g., simple RT, visual search, attentional selection), and as noted by Toth et al. (2020), predictive timing tasks remain underrepresented—likely because predictive timing is not a central, explicitly trained component of most FPS gameplay.

Given these task characteristics, it is plausible that FPS-specialized players prioritize rapid stimulus–response execution over fine-grained temporal calibration during prediction-based tasks. Accordingly, the absence of a group difference in RE suggests that FPS experience may not confer a broad transfer advantage in temporal prediction precision.

By contrast, the reduced PE at response observed in the FPS-specialized group indicates a more accurate spatial prediction of the target arrival point. Prior evidence suggests that extended FPS engagement enhances visuospatial processing (Kokkinakis et al., 2017) and that spatial indices are predictive of FPS performance (Warburton et al., 2023). From this perspective, enhanced spatial prediction—specifically, target-arrival prediction—appears to represent a key cognitive strength of FPS-specialized players.

Taken together, these findings suggest that the smaller PE values observed in the FPS-specialized group likely reflect a superior ability to rapidly infer spatial action goals from visual information and efficiently translate those predictions into motor output. Thus, within the context of this task, spatial precision—not temporal precision—emerged as the primary dimension along which FPS-specialized players demonstrated an advantage.

#### 4.1.2 Time-series dynamics

Time-series analyses revealed that the FPS-specialized group consistently maintained a smaller PE and fixated closer to the target’s final landing position than the non-FPS group, beginning at approximately 0.50 s and continuing until the end of the trajectory 4.60 s. Significant group differences in PE emerged during 0.50–0.62 s, 0.66–0.76 s, 1.02–2.18 s, and 2.56–4.60 s. Likewise, horizontal eye position differed across the entire 0.50–4.60 s interval, with the FPS-specialized group shifting gaze toward the predicted arrival position earlier and maintaining fixation closer to that location. These findings indicate that the FPS-specialized group shifted gaze toward the anticipated arrival position substantially earlier than the non-FPS group.

Group differences were also observed in vertical eye position. The non-FPS group showed significantly greater upward deviations during 1.30–1.92 s and 2.66–2.70 s. Notably, the FPS-specialized group exhibited only a minimal vertical gaze elevation, with the highest gaze position reaching 905.80 pixels at 0.90 s (approximately 1.83° above the initial target position). This minimal elevation suggests that, FPS-specialized participants derived sufficient information from initial motion parameters— velocity and direction—without requiring visual tracking of the full parabolic arc. The brief vertical difference observed at 2.66–2.70 s, despite only modest differences in mean gaze positions between groups, likely reflects reduced interindividual variability in the non-FPS group rather than a systematic strategy difference, as suggested by time-series distribution patterns.

In contrast, the non-FPS group exhibited significantly smaller PE during the early interval (0.06–0.26 s) and positioned their horizontal gaze closer to the eventual landing point during 0.04–0.26 s. These early differences appear to reflect pre-movement fixation strategies rather than prediction accuracy. Horizontal gaze patterns indicated that the FPS-specialized group maintained fixation near the target center (164.94 pixels), whereas the non-FPS group positioned their gaze slightly forward relative to the target’s path (181.06 pixels). Vertically, the non-FPS group held gaze approximately 34.73 pixels (0.97°) above the target. In other words, the non-FPS group adopted a forward-biased pre-movement fixation aligned with the expected direction of motion.

This strategy is disadvantageous for accurate motion extraction motion. When a moving object approaches the fovea, the magnitude of the flash-lag effect increases, leading to a perceptual experience in which the moving object appears ahead of its true position (Linares and Holcombe, 2008). Thus, by fixating ahead of the moving target, the non-FPS group likely entered a perceptual regime in which the target appeared displaced forward in space. This perpetual bias delays accurate motion extraction, delaying the formation of a spatial prediction and ultimately manifesting as the sustained PE differences observed from 0.50–4.60 s.

The FPS-specialized group, by contrast, maintained central fixation on the target at motion onset and rapidly extracted velocity–direction information before executing an anticipatory gaze shift toward the occlusion boundary. Such anticipatory gaze strategies have been reported as a hallmark of high-level FPS performance. For example, Jeong et al. (2025) demonstrated that highly skilled FPS players exhibit sharper attentional focus and reduced gaze dispersion under selective demand, facilitating faster visuomotor responses.

A key mechanism underlying this behavior is the superior spatial resolution of foveal vision, which is essential for detecting fine-grained, continuous positional changes—such as those encoding motion direction and speed—in the present task (e.g., Robinson, 1965). By maintaining foveal fixation on the target’s center at motion onset, the FPS-specialized group was able to extract accurate motion parameters from a very early phase of the trajectory, enabling them to shift gaze toward the predicted landing position at an unusually early stage.

Collectively, these findings reveal a fundamental strategic divergence between groups. The FPS-specialized group relied on anticipatory gaze control, whereas the non-FPS group relied on a forward-biased, misaligned pre-movement fixation strategy that delayed accurate spatial prediction. This strategic difference explains the sustained PE separation, the earlier and more precise fixation of the predicted arrival position, and the overall performance advantage shown by the FPS-specialized group across the time series.

### 4.2 Transition to neural mechanisms of predictive gaze control

Although the eye-position results clearly demonstrate that the FPS-specialized group relied on rapid extraction of motion information and early anticipatory gaze shifts toward the predicted arrival position, behavioral data alone cannot determine the underlying neural mechanisms supporting this strategy. Anticipatory gaze control requires internal spatial prediction, visuomotor transformation, and motor preparation—even when visual information is no longer available. To identify the neural processes supporting these predictive gaze strategies, the following section examines the EEG activity recorded during the task and evaluates how cortical dynamics differed between the FPS-specialized and non-FPS groups.

### 4.3 Electroencephalography

Because EEG analyses were time-locked to stimulus onset rather than saccade onset, the observed neural activity is interpreted as reflecting predictive visuomotor planning and internally guided saccade-related processes rather than movement execution per se. This distinction is particularly relevant, given the anticipatory gaze strategy adopted by the FPS-specialized group.

#### 4.3.1 Occipital cortex (BA18; O2): visual imagery and predictive maintenance

Electrode O2 corresponds primarily to Brodmann area (BA) 18, located within the middle occipital gyrus, based on probabilistic Talairach mapping (*x* = 25.0 ± 5.7 mm, *y* = −95.2 ± 5.8 mm, *z* = 6.2 ± 11.4 mm) reported by Koessler et al. (2009). In the FPS-specialized group, significant low-beta activity at O2 was observed during two brief intervals (1.814–1.830 s and 1.843–1.862 s) following motion onset. At these time points, gaze had already shifted toward the predicted arrival position (PE at 1.82–1.86 s = approximately 2.14°; PE at response = 2.00°), indicating that spatial prediction was already complete and that participants had transitioned into a response preparation phase. In contrast, the non-FPS group showed significant O2 activation much earlier (0.890–0.908 s and 0.925–0.941 s). During these intervals, gaze remained near the occlusion boundary (horizontal gaze position at 0.90–0.94 s = 628.82–646.89 pixels; occlusion onset = approximately 613 pixels) rather than at the predicted arrival position.

BA18, corresponding largely to the secondary visual cortex (V2), contributes to higher-order visual processing, —including the refinement of motion and shape information, visual attention support, and visual imagery or predictive representation (Dijkstra et al., 2017). This functional profile suggests that BA18 participates not only in bottom-up sensory processing but also in the reactivation of stored visual information to support prediction of future perceptual states. Within this framework, the later BA18 activation observed in the FPS-specialized group likely reflects the strategic maintenance of a predicted visual outcome—that is, sustaining an internal image of the target location after gaze has shifted away. This interpretation aligns with Jeong et al. (2025), who reported that highly skilled FPS players exhibit enhanced capacity to sustain attention and maintain stable fixation on task-relevant goals.

In the non-FPS group, however, BA18 activation emerged earlier, when gaze had not yet reached the predicted arrival position. Their gaze pattern suggest incomplete trajectory tracking and an absence of fixation on the landing position during these periods. Thus, early BA18 activation in the non-FPS group may reflect visual imagery or perceptual maintenance of the last seen target state, rather than updated predictive representation. Such maintenance is adaptive only when the predicted arrival position has already been localized—which was not the case for this group. As a result, cognitive resources in the non-FPS group were likely allocated toward maintaining the visual representation rather than updating predicted motion, limiting engagement of predictive visual, visuomotor, and motor planning processes. This imbalance would favor static visual maintenance over dynamic prediction and may explain the delayed formation of an anticipatory gaze strategy and the larger PE at response observed in this group.

#### 4.3.2 Parietal cortex (BA7; CP2/P4): spatial prediction and coordinate-based representation

Electrodes CP2, P3, and P4 correspond primarily to BA7, based on the probabilistic atlas of Koessler et al. (2009). Specifically, CP2 is located within the postcentral gyrus (*x* = 25.8 ± 6.2 mm, *y* = −47.1 ± 9.2 mm, *z* = 66.0 ± 7.5 mm), P3 within the inferior parietal lobule and precuneus (*x* = −41.4 ± 5.7 mm, *y* = −67.8 ± 8.4 mm, *z* = 42.4 ± 9.5 mm), and P4 within the inferior parietal lobule (*x* = 44.2 ± 6.5 mm, *y* = −65.8 ± 8.1 mm, *z* = 42.7 ± 8.5 mm). Collectively, these locations indicate that the electrodes sampled activity within the posterior parietal cortex, a region central to visuospatial processing and sensorimotor transformation. In the FPS-specialized group, significant BA7 activity emerged at CP2 during 0.554–0.576 s and 0.589–0.607 s, and at P4 during 0.567–0.579 s and 0.602–0.619 s following motion onset. At these times, gaze had already shifted toward the occlusion region (horizontal gaze position at 0.56–0.62 s: 722.58–772.28 pixels; occlusion onset = approximately 613 pixels). Thus, this neural activity likely reflects predictive visual processing based on previously extracted motion information, rather than processing of visible stimuli. In contrast, the non-FPS group showed bilateral BA7 activation at earlier time windows (CP2: 0–0.018 s. 0.043–0.131 s; P3: 0.034–0.065 s), likely reflecting a reactive response to the sudden onset of motion rather than engagement in spatial prediction.

The functional role of BA7 aligns with its contribution within the dorsal visual stream, where it transforms external spatial input into body-centered coordinate representations to support goal-directed action (Cooper and O'Sullivan, 2016). BA7 is particularly associated with encoding object location and establishing egocentric reference frames for eye and reaching movements (Baumann and Mattingley, 2014). Evidence from lesion research demonstrates that impairments to the parietal cortex disrupt spatial relation tasks in rodents (Rogers and Kesner, 2006), supporting its role in maintaining and manipulating spatial information. Complementary neuroimaging work by Renier et al. (2009) demonstrated activation in the right parietal cortex (BA7/BA40) during multisensory “Where” tasks involving auditory and tactile cues, indicating that BA7 participates in multisensory spatial representation.

Taken together, the observed BA7 activation in the FPS-specialized group likely reflects the integration of visual motion cues to generate an internal spatial prediction once visual input is withdrawn via the predictive saccade strategy. This neural pattern is consistent with formation of a coordinate-based spatial goal, which guides gaze and attention toward the predicted arrival position.

By contrast, early bilateral BA7 activity in the non-FPS group may reflect categorical spatial encoding driven by stimulus onset rather than predictive mapping. Baumann and Mattingley (2014) proposed hemispheric specialization within BA7: the right hemisphere preferentially encodes continuous metric spatial representations, whereas the left hemisphere supports categorical relational encoding. Applied here, the right BA7 activity in the FPS-specialized group likely reflects the construction and maintenance of a precise spatial estimate derived from motion cues, enabling fine-grained prediction of target arrival. In contrast, left-lateralized or early bilateral activity in the non-FPS group likely reflects categorical, stimulus-driven encoding rather than motion-informed spatial prediction. In summary, although both groups engaged BA7 during the task, their neural signature diverged. The non-FPS group appeared to rely on early categorical spatial encoding following motion onset, whereas the FPS-specialized group recruited BA7 during a later phase of the trajectory, relative to the non-FPS group, to support precise, motion-informed coordinate spatial representations, enabling proactive, predictive gaze behavior.

#### 4.3.3 Supramarginal gyrus (BA40; CP5): multisensory integration and predictive visuomotor transformation

Electrode CP5 corresponds to BA40 based on Talairach coordinates (*x* = −61.8 ± 4.7 mm, *y* = −46.2 ± 8.0 mm, *z* = 22.5 ± 7.6 mm) and is situated within the supramarginal gyrus (SMG; Koessler et al., 2009). In the FPS-specialized group, significant BA40 activity emerged during 0.554–0.579 s following motion onset. At this time, gaze had already shifted toward the region where the target would later become invisible due to the anticipatory saccade strategy (horizontal gaze position at 0.56–0.58 s: 722.58–741.81 pixels; occlusion onset = approximately 613 pixels). Thus, this neural response cannot be attributed to processing of visible stimuli and instead reflects predictive visuomotor processing based on previously extracted motion information.

BA40—particularly the SMG—is widely recognized as a core hub for multisensory integration and visuomotor transformation. Lee Masson et al. (2016) demonstrated selective activation of left BA40 during convergence of visual and tactile shape information, suggesting that BA40 supports the formation of modality-independent shape representations that can guide action. From this perspective, the left BA40 activation observed here likely reflects the transformation and maintenance of recently acquired visual information into an action-usable, body-centered representation, supporting internal updating of the predicted saccade target under conditions where visual input is intentionally not absent. In this context, the activity reflects not passive visual maintenance but a component of predictive visuomotor computation required to support forthcoming movement preparation.

Further support comes from Budisavljevic et al. (2017), who identified robust functional coupling between the left BA40 (SMG) and BA6 (premotor cortex) during reach-planning tasks. Their findings suggest that BA40 translates perceptual spatial goals into motor program coordinates and supports left-hemisphere specialization for sequential action planning. This framework aligns with Baravalle et al. (2019), who observed BA40–BA6 coupling during both executed and imagined movements, indicating a role in predictive motor control, including specifying *how* and *when* an action should be initiated. These predictive mechanisms are believed to generalize to gaze control, offering a plausible neural basis for predictive saccade control.

Collectively, these converging findings suggest that BA40 functions as an integrative hub coordinating spatial prediction and emerging motor plans during predictive saccade control. In the present study, left BA40 likely served as a linking node, enabling communication between right BA7, which provides the spatial “where” component of the predicted landing position, and left BA6, which computes the motor “how/when” parameters of the forthcoming saccade. Through this coordinated interaction, BA40 supported predictive visuomotor transformation during anticipatory gaze control under conditions in which visual input was intentionally unavailable due to the saccade strategy.

#### 4.3.4 Premotor cortex (BA6; FC1): predictive motor planning and internally generated visuomotor commands

According to Koessler et al. (2009), electrode FC1 corresponds primarily to BA6 within the superior frontal gyrus (*x* = −24.7 ± 5.7 mm, *y* = 0.3 ± 8.5 mm, *z* = 66.4 ± 4.6 mm). In the FPS-specialized group, significant BA6 activation occurred during 0.603–0.615 s after motion onset. At this interval, gaze had already shifted toward the visually unavailable region due to the predictive saccade strategy (horizontal gaze position at 0.60–0.62 s: 758.42–772.28 pixels; occlusion onset = approximately 613 pixels). Thus, this activation is unlikely to reflect processing of currently available visual input and instead indicates engagement of predictive motor planning based on previously acquired visuomotor information.

Dorsal BA6 has been consistently associated with motor planning and temporal control of actions derived from sensory input. Extensive evidence (Cisek and Kalaska, 2010; Hoshi and Tanji, 2004) demonstrates that BA6 plays a key role in determining *when* and *in which* direction an action should be initiated, functioning in coordination with posterior parietal regions—particularly BA7—to transform sensory information into motor representations. BA6 contributes to the formulation of internal motor programs prior to movement execution and has been linked to preparatory neural activity underlying reaching movements and predictive saccades.

In the present study, the timing of BA6 activity—after the initial saccade had already been executed but before fixation reached the predicted arrival position—suggests reliance on internally maintained visuomotor representations. Because the task design intentionally prevented online visual guidance, BA6 activation is best interpreted as reflecting fine-tuning of the ongoing saccade sequence rather than the initiation of the initial gaze movement. In the FPS-specialized group, this profile indicates the generation of an internally driven predictive motor command rather than a reactive response to new visual input. Moreover, the temporal characteristics of BA6 engagement parallel prior observations that BA6 operates jointly with BA40 during predictive visuomotor control (Baravalle et al., 2019; Budisavljevic et al., 2017). In this framework, BA6 refines the motor “how/when” parameters of the emerging saccade based on the spatial “where” information encoded by BA7 and the integrative mapping performed by BA40. Thus, BA6 activity in the FPS-specialized group reflects a coordinated neural mechanism supporting predictive saccade execution under conditions in which visual input is intentionally absent due to the task strategy.

#### 4.3.5 Coordinated engagement of BA7, BA40, and BA6 supporting predictive saccade control in the FPS-specialized group

In the FPS-specialized group, three regions—the right BA7 (posterior parietal cortex), left BA40 (SMG), and left BA6 (premotor cortex)—showed temporally clustered activation within a narrow window (0.554–0.619 s) following target motion onset. As described in preceding sections, right BA7 activity reflects spatial encoding of the target motion and the formation of a coordinate-based internal estimate of the predicted arrival position, specifying where attention and subsequent gaze should be directed. In parallel, left BA6 activity indexes the generation of an internally driven predictive motor command that supports preparation and temporal refinement of the predictive saccade. Left BA40, in turn, appears to function as an integrative hub, linking the spatial estimate supplied by parietal regions (e.g., BA7) with the developing motor plan within premotor regions (e.g., BA6), facilitating their coordinated engagement rather than functioning in isolation.

The concurrent recruitment of these regions suggests that the FPS-specialized group relied on a coordinated visuomotor prediction network in which predictive saccade control was guided by internally maintained visuomotor representations rather than by continuous visual monitoring. This interpretation aligns with prior evidence demonstrating functional interdependence across these areas. Lee Masson et al. (2016) demonstrated cooperative activation between BA7 and BA40 during visuohaptic processing, supporting their roles in constructing modality-independent spatial representations. This relationship is consistent with the present interpretation that BA7–BA40 coupling may support the transformation of predicted spatial goals into actionable visuomotor representations. Further supporting this network interpretation, Budisavljevic et al. (2017) showed strong functional coupling between left BA40 and BA6 during reach planning, highlighting their contribution to motor sequencing. Baravalle et al. (2019) likewise observed BA40–BA6 interaction during both executed and imagined movements, implicating the SMG in predictive motor control. Complementarily, research by Cisek and Kalaska (2010) and Hoshi and Tanji (2004) emphasizes interactions between BA6 and posterior parietal regions, including BA7, during the conversion of sensory information into motor representations. Taken together, these findings support the interpretation that right BA7, left BA40, and left BA6 operated as a bidirectionally coordinated visuomotor network in the FPS-specialized group, enabling motor timing, predictive saccade preparation, and efficient execution.

Prior literature in action gaming suggests that experienced VGPs show enhanced suppression of irrelevant information and more efficient allocation of visual attention (Bavelier et al., 2012a, 2012b; Mishra et al., 2011). These characteristics align with the present observation that the FPS-specialized group relied less on early visual input—as observed in the non-FPS group—and instead used strategies centered on internal updating and predictive planning, enabling gaze shifts toward the predicted arrival position earlier in the trajectory.

In summary, the present findings indicate that predictive saccade control in the FPS-specialized group reflects the coordinated engagement of a functionally integrated visuomotor network: right BA7 provides spatial prediction and goal-related representation, left BA6 generates internally driven motor preparation commands, and left BA40 links these cognitive and motor processes. Together, these regions support internally guided visuomotor transformation, enabling rapid and accurate predictive saccades despite the intentional absence of ongoing visual input imposed by the saccade strategy.

### 4.4 Limitations

This study has several limitations that should be acknowledged.

First, although the FPS-specialized group consisted of individuals with verified competitive experience, the degree of competitive involvement within the group remained heterogeneous and the overall sample size was modest. Although participants were categorized based on competitive engagement rather than gameplay duration, objective continuous measures, such as hours of play per week or ranking-based metrics were not uniformly available and therefore could not be incorporated as covariates in the present analyses. While this classification aligns with the theoretical aim of distinguishing competitively engaged FPS players from those without tournament experience, variability in competitive exposure limits the precise dissociation of FPS-specific expertise from broader action video game experience and may constrain generalizability. In addition, the present sample was predominantly male, which further limits generalizability. Previous neuroimaging studies have discussed the possibility of sex-related differences in visuomotor engagement and network-level neural plasticity in action VGPs (e.g., Cahill and Dhamala, 2025). Future studies with more balanced samples will be necessary to directly address potential sex-related effects. Nonetheless, the consistent behavioral and neural differences observed between the FPS-specialized and non-FPS groups, even within a heterogeneous competitive sample, suggest that the identified visuomotor and predictive control characteristics are robustly associated with competitive FPS engagement. Future research should examine whether these neural signatures extend across sexes and to elite esports professionals competing at the highest international levels.

Second, although the present study relied on probabilistic electrode–to–region mapping (Koessler et al., 2009), scalp EEG does not allow precise source localization. The BA labels used here should therefore be interpreted as the most probable cortical associations rather than precise anatomical identifications. This limitation reflects both the relatively low spatial resolution of EEG and the limited electrode count of the current setup. Future work employing high-density EEG, source reconstruction methods (e.g., standardized low-resolution brain electromagnetic tomography (sLORETA), beamforming), or multimodal neuroimaging (e.g., EEG–fMRI) could refine localization and validate the proposed regional interpretations.

Third, owing to the visuomotor nature of the task, systematic eye movements constitute an essential component of performance and cannot be fully dissociated from EEG signals. Therefore, EEG analyses were designed to emphasize sustained, stimulus-locked, group-level modulation of low-beta amplitude envelopes associated with predictive visuomotor processing, rather than transient neural activity time-locked to individual eye movements. Future studies with larger samples and higher-density recordings may further dissociate oculomotor and neural contributions using advanced artifact modeling and source-resolved approaches.

Fourth, EEG analyses were restricted to the visible phase of the trajectory and did not include the occluded period during which predictive maintenance and internal simulations were theoretically expected to be prominent. This decision was motivated by the experimental design in which the occluded phase frequently overlapped with button-press responses and associated motor preparation, increasing the risk of contamination by movement-related neural activity. As a result, the present EEG findings primarily characterize predictive visuomotor processes during the visually guided phase rather than neural dynamics underlying prediction maintenance in the absence of visual input. Future studies employing response-delayed or motor-decoupled paradigms are needed to directly examine neural mechanisms that support prediction during occlusion.

Fifth, the eye-tracking system operated at a moderate sampling rate (50 Hz), which did not permit direct estimation of precise saccade onset latency, microsaccades, or very fine-grained oculomotor dynamics. Consequently, the present gaze analyses were designed to characterize group-level predictive gaze dynamics inferred from gaze position and PE time courses, rather than to resolve precise oculomotor timing parameters. In addition, gaze time-series data were analyzed using pointwise independent-samples *t*-tests primarily to visualize the temporal evolution of group differences. Although FDR correction was applied, this approach did not explicitly model the temporal autocorrelation inherent in eye-position signals. Accordingly, continuous intervals of significance should be interpreted as reflecting sustained group-level differences in predictive gaze behavior rather than sharply localized temporal effects. Future studies with larger sample sizes may benefit from time-series modeling approaches that directly account for temporal dependence.

Finally, the EEG analysis focused exclusively on the low-beta band (12–16 Hz) to maintain methodological continuity with prior work. Future research may benefit from broader spectral characterization or connectivity-based approaches (e.g., phase synchronization, Granger causality, dynamic causal modeling) to capture the temporal coordination among BA7, BA40, and BA6 during predictive saccade control.

## 5 Conclusion

The present study demonstrated that FPS-specialized players employ a distinct predictive saccade strategy supported by enhanced visuospatial processing and temporally organized neural activity. Behaviorally, the FPS-specialized group exhibited superior spatial prediction accuracy, despite no group differences in temporal estimation, indicating a selective advantage in estimating the target landing position. Time-series analyses of gaze position further showed that FPS-specialized players extracted motion information more rapidly and shifted their gaze earlier and more precisely toward the predicted arrival location.

Neurally, the analysis of low-beta activity identified three key cortical regions— the right BA7, left BA40, and left BA6—that were selectively engaged within a narrow temporal window during the visible portion of the trajectory. The right BA7 contributed to spatial prediction and coordinate-based representation of the target, the left BA6 supported predictive motor planning for the upcoming saccade, and the left BA40 functioned as an integrative hub linking spatial estimation with emerging motor commands. The coordinated engagement of these regions suggests the recruitment of an internally driven visuomotor transformation process that underlies the early and accurate predictive saccades observed in FPS-specialized players.

Together, these findings demonstrate how domain-specific experience in FPS gameplay shapes predictive gaze strategies and their supporting neural mechanisms. By illustrating how players form internal spatial predictions and convert them into anticipatory visuomotor actions, this work advances understanding of predictive control in mediated, dynamic, and interactive environments. These insights contribute to broader research on embodied perception, interface-driven skill acquisition, and the neural basis of sensorimotor expertise in virtual and technologically mediated contexts.

## Data Availability

The raw data supporting the conclusions of this article will be made available by the authors, without undue reservation.
